# Antioxidant and Biological Properties of Mesenchymal Cells Used for Therapy in Retinitis Pigmentosa

**DOI:** 10.3390/antiox9100983

**Published:** 2020-10-13

**Authors:** Paolo Giuseppe Limoli, Enzo Maria Vingolo, Celeste Limoli, Marcella Nebbioso

**Affiliations:** 1 Low Vision Research Centre of Milan, p. Sempione 3, 20145 Milan, Italy; celeste.limoli@libero.it; 2 Department of Ophthalmology, A. Fiorini Hospital, Terracina, Polo Pontino, Sapienza University of Rome, p. le A. Moro 5, 00185 Rome, Italy; evingolo@libero.it; 3 Department of Sense Organs, Faculty of Medicine and Odontology, Sapienza University of Rome, p. le A. Moro 5, 00185 Rome, Italy; marcella.nebbioso@uniroma1.it

**Keywords:** retinitis pigmentosa, MSC, cell therapy, oxidative stress

## Abstract

Both tissue repair and regeneration are a priority in regenerative medicine. Retinitis pigmentosa (RP), a complex retinal disease characterized by the progressive loss of impaired photoreceptors, is currently lacking effective therapies: this represents one of the greatest challenges in the field of ophthalmological research. Although this inherited retinal dystrophy is still an incurable genetic disease, the oxidative damage is an important pathogenetic element that may represent a viable target of therapy. In this review, we summarize the current neuroscientific evidence regarding the effectiveness of cell therapies in RP, especially those based on mesenchymal cells, and we focus on their therapeutic action: limitation of both oxidative stress and apoptotic processes triggered by the disease and promotion of cell survival. Cell therapy could therefore represent a feasible therapeutic option in RP.

## 1. Introduction

Retinitis pigmentosa (RP) affects 1.5 million people around the world, representing the most widespread hereditary retinal dystrophy: globally, its prevalence is estimated at 1:4000.

The term ‘RP’ comprises a series of clinical conditions caused by a high number of genetic alterations that, either alone or in association, cause damage to the molecular processes necessary for the creation, conservation, use, or recovery of rhodopsin. The direct consequence is the progressive and total loss of rod cells [1,2,3].

The genetic etiology of RP underlies the damage and subsequent death of rod cells, while the central retina, which contains mainly cone cells, remains in relatively good condition until the advanced stage of the disease. This explains why RP patients are often diagnosed later on in life, after the second or third decade of life.

However, the clinical manifestations of RP are caused not only by rod cell loss but also by the cone cell injury, albeit in later phases.

The cone loss goes beyond genetics [4,5,6] and involves other biomolecular mechanisms, including alterations in hemodynamics [7], oxidative stress due to the higher availability of oxygen after rod loss [8,9], and the impaired response to oxidative stress [2,3,10,11,12].

This sequence of events underlies the prevailing symptoms of RP: night blindness, tunnel vision, followed by progressive loss of central vision and complete or near complete blindness.

Rod cells account for about 95% of all photoreceptors, and the oxidative metabolism of fatty acids is their main source of energy [13].

More than 80 causative genes of RP responsible for rod damage have already been identified, although a significant number of them are still unknown [14].

Genetic mutations responsible for RP in some cases also involve genes expressed not only in rods but also in the retinal pigment epithelium (RPE), such as MERTK [15], RLBP1 [16], and RPE65 [17].

RPE plays many vital roles for photoreceptor cells, and the most fascinating is certainly its protective action against oxidative stress [18].

Recent studies have confirmed a high level of reactive oxygen species (ROS) in RPE, and fatty acids are one of their molecular targets. If oxidized, they can compromise transduction pathways and gene expression [19].

At this point, a cascade of molecular phenomena—such as para-inflammation, synaptic impairment, apoptosis, and cell death—which hugely impact visual function, is triggered.

Therefore, oxidative damage is considered the leading cause of cone apoptosis and progressive vision loss [6,7,20,21].

However, this chain of events, which is triggered after the rod death and leads to the cone loss, highlights a number of key points that can potentially be leveraged therapeutically to slow down or stop the disease progression towards its terminal stages, modulating the rod damage and preventing or delaying cone death [22,23,24].

In order to stimulate neuronal survival, many research groups have worked on animal models of RP.

New therapeutic approaches for RP include the restoration of defective genes and stem cell transplantation to replace or repair impaired or dead cells [25,26].

## 2. Oxidative Stress and Retinitis Pigmentosa

### 2.1. Animal Models of RP

There are a complex variety of animal models that have allowed the molecular study of RP.

The refinement of these genetic models offers a deeper comprehension of biological and etiopathogenetic mechanisms of the disease. Based on these studies, it is also possible to develop new treatments and prevention strategies.

Examples of those models are Rd1 mices [27], Rd10 mices [28], P23H and S334ter Rhodopsin Transgenic Rats [29], Rd mices [30], Rds mices [31], Royal College of Surgeons rats [32], and RPE65 dog [33].

Rd1/rd1 mouse has a mutation at the level of β subunit of phosphodiesterasis cGMP gene that leads to cGMP toxic accumulation, higher level of intracellular Ca2, and finally rod death [27,34,35,36,37]. The rod loss leads to a greater amount of oxygen available, that injures the cones, causing their death. In view of this, antioxidative therapy could prevent cone death in this RP murine model [34,35,36,37].

A similar mutation has been found in a particular type of autosomal recessive RP, and therefore Rd1/rd1 mouse has become an ideal RP model [34].

Rd10 mouse has allowed the study of ceramide in retinal degeneration. Ceramide is a proapoptotic sphingolipid and its level increases during the rod cell death.

It has been shown that the photoreceptor loss can be blocked by hindering the ceramide proapoptotic pathway.

Intraocular injection or continuous eye drops administration of myriocin, inhibitor of serin palmitoil-CoA transferase, can return ceramide to normal levels and stop the apoptotic death of photoreceptors. Therefore, this therapeutic approach can be applied to humans [28].

P23H rat model has established that the photoreceptor loss triggers major changes in the number and morphology of glial cells affecting the inner retina.

Both astrocytes and Müller cells promote retinal cell survival by releasing neurotrophic factors, providing anti-oxidative support, catabolizing neurotransmitters in the extraneural space, and supporting synapse formation. They also contribute to activating microglial cells and regulating vasal tone [38]. In addition to the photoreceptor loss in P23H rat model, the alteration of retinal vascular plexuses has been observed. The reduced capillary density may hinder the oxygen and nutrient supply to the retinal cells and foster the retinal degeneration. Thus, vascular injuries should be considered as an important therapeutic target in degenerative retinal diseases [39].

In Rd [30], Rds [31], in Royal College of Surgeons rat [32], and in RPE65 dog [33], the identification of a single mutation has allowed to develop targeted gene therapy and to partially limit the retinal degeneration. However, there are only few types of RP with specific mutations, restricting the application of gene therapy.

The use of trophic factors [40,41,42], calcium channel blockers [43], or MSCs [44] have been observed to slow down the disease progression in some RP animal models.

### 2.2. Synoptic Aspects of Oxidation and Antioxidation

Photoreceptors are particularly sensitive to oxidative damage exerted by the light, with which they constantly interact [45,46,47]. In fact, to phototransduce electromagnetic radiation into visual stimuli, retinal cells contain numerous photosensitive molecules, a considerable amount of polyunsaturated fatty acids (15% of photoreceptor’s mass) and are characterized by an extremely high metabolism, from which unstable metabolic byproducts, called ROS, are continuously generated. ROS are represented by several unstable molecules, including superoxide anion (O_2_^−^), ozone (O_3_), hydrogen peroxide (H_2_O_2_), hydroxyl radical (^•^OH) derived from the decomposition of peroxides, peroxide radical (LOO·) which removes an atom of hydrogen from another lipid molecule, and nitric oxide (NO·), a messenger in many cytosolic pathways.

Furthermore, under oxidative stress conditions, non-metabolizable advanced glycation end-products (AGEs), responsible for para-inflammation and permanent cell damage, are produced [48].

Over time, oxidative stress can alter transduction pathways and gene expression [49] and damage all the cellular components, including phospholipid membranes, proteins, and nuclear and mitochondrial DNA (mtDNA). Those injuries lead to the progressive loss of function of photoreceptor as well as RPE [50,51,52].

However, photoreceptors are able to protect themselves against these oxidative injuries through several mechanisms.

The first antioxidant defense is mediated by enzymes—such as catalase, glutathione peroxidase, and reductase—which promote the decomposition of hydrogen peroxide into water and oxygen molecules; superoxide dismutase (SOD), which is normally found in the mitochondria of cone’s inner segments [53,54,55] or the glyoxalase system [56], which neutralizes ROS by acquiring electrons from oxidizing substances.

Another important defense is provided by the endoplasmic reticulum (ER) through the activation of a cellular stress response, called unfolded protein response (UPR). As a reaction to the accumulation of misfolded proteins in the ER lumen, UPR is initially set to restore normal cell function; if this process does not occur in the proper time and way, UPR activates apoptosis. The persistent activation of UPR has been implicated in the pathogenesis and progression of several diseases, such as RP [57,58].

Another protective mechanism against oxidative stress is the production of stress granules, proteins able to bind and protect specific mRNAs, preventing their degradation. Through the selective inhibition of such mRNAs, the transcription of constituent genes is selectively blocked while the translation of stress-induced transcripts is facilitated, allowing energy savings and cell survival [59].

Furthermore, retinal cells can resort to autophagy to catabolize damaged proteins and organelles, ensuring a homeostatic balance and promoting their survival following oxidative damage [11,60].

About 1–5% of ROS is generated in the mitochondria, organelles responsible for energy production in the cell. As a response to specific signals including oxidative stress, hunger, and mitochondrial protein modification, the selective autophagy of mitochondria can be activated [61].

Autophagy plays a protective role against oxidative stress and other cellular lesions, but the build-up of autophagosomes due to prolonged insults ends up becoming harmful to cells [62].

Finally, RPE cells have been shown to protect photoreceptors against ROS [63]. They are also known to provide many other vital functions for photoreceptors, such as light absorption, bi-directional epithelial transport, spatial ion buffering (in order to maintain the predisposition to depolarization), visual cycle regulation, phagocytosis of external photoreceptor segments (POS), secretion of trophic factors and signaling molecules, and support to the eye seen as an immunologically privileged site [64].

In conclusion, the balance between oxidative stress and antioxidant mechanisms is crucial for cell survival. If the cell is over-stressed or has an altered protection (e.g., due to pathologies), programmed death cell, i.e., apoptosis, is induced [65,66,67,68,69]. Therefore, it is necessary to preserve the homeostasis to avoid cell death by regulating the excess of ROS that the metabolism continuously produces.

It is especially true for RP in which the impairment of antioxidant responses has a key role in triggering the disease progression [12,47].

In fact, the photoreceptors—in particular the rods, responsible for scotopic vision, and the RPE—are the most vulnerable cell types to oxidative damage [3], especially because they are believed to reside in a terminal G0 phase.

### 2.3. Oxidative Stress and RP

The impairment of retinal vascularization, mainly mediated by oxidative stress, is considered to play a key role in the RP progression.

Many studies have shown a reduction both in choroidal [61,70] and macular [4,71] hemodynamics associated with a reduced visual sensitivity in RP patients.

The catabolic products released by photoreceptors not only lead progressively to rod loss but also have negative effects on microcirculation. In fact, the retinal vessels appear thin. It becomes a vicious circle in which the altered perfusion fosters photoreceptor injury and loss [72].

Several studies highlight the role of the impaired retinal circulation in RP and its correlation with residual function [73] and choroidal thickness [74]. In particular, the reduction in retinal blood flow both as a whole [20] and at the subfoveal level has been shown, with related alterations in electroretinographic recordings [75].

Several studies have shown that both endogenous ROS produced by retinal metabolism and the lipid peroxidation or DNA damage, produced by external agents, such as exposure to sunlight or cigarette smoke, can contribute to photoreceptor death.

The most pathognomonic aspect of RP is that the blood, passing through the choroid, maintains an arterial oxygen saturation until it enters the venous system. Moreover, unlike retinal capillaries, choroidal capillaries allow plasma protein diffusion in order to meet the metabolic photoreceptor needs [76].

The rods, which make up about 95% of all photoreceptors, are progressively lost in RP; consequently, the intracapillary oxygen level remains elevated, increasing ROS production and inducing an oxidative damage in the cones, surviving cells that are eventually impaired and lost [9,45].

The following factors have been shown to exacerbate the oxidative damage and the rod death: foveal area’s exposure to light, choroidal stasis, metabolic deterioration of cones and RPE cells, lack of antioxidant enzymes such as SOD, which is normally found in the mitochondria of the cone inner segments (but not in the outer ones), glutathione peroxidase, glyoxalase and catalase, and autophagy impairment [8,10,11,77,78,79].

In recent years, it has been demonstrated that the oxidative damage can also interfere with particular RNA molecules called long non-coding RNAs [80,81]. These are involved in several critical biochemical pathways, such as chromosome conformation modeling, genomic imprinting modulation, allosteric control of enzymatic activity, as well as cell state coordination, differentiation, and development. Dysregulation or mutation of non-coding genes has been associated with various human diseases, including RP [80,81].

The alteration of lipoproteins and DNA derived from hyperoxia can cause irreparable damage in the residual cells (mainly cones), and therefore in the foveal region [9,45,79,82,83,84].

In RP, the cell apoptosis induced by oxidative stress determines the so-called retinal gliosis, i.e., a state of para-inflammation in which microglial and macroglial cells are activated [85].

The microglial cells, which are normally dormant resident retinal macrophages, provide neuroprotection against ROS damage under physiological conditions.

Debris from apoptotic or dead cells, damaged lipopolysaccharides and ROS [21,86] can trigger the activation of apoptotic photoreceptors in RP, which generally occurs just before or at the peak of apoptotic photoreceptor death [87,88,89].

Their activation involves the expression of inflammatory regulatory proteins such as peroxiredoxin 2 (PRDX2), pro-inflammatory cytokines such as TNF-α, interleukin-1β or interferon-γ in RPE cells [90,91], chemokines and neurotoxic agents, including hydrogen peroxide, and superoxide anion with additional oxidative stress [92,93].

The microglia chronic activation promotes the microglial phagocytosis against the altered components of neuronal cells, determining the evolution of RP [94].

Conversely, the suppression of their activation improves the survival of rods [95].

On the other hand, the macroglia represented by retinal Müller glia (RMG)—which form the columns of retinal tissue and have multiple connections with retinal neurons, microglia, astrocytes, and endothelial cells—modulate different responses depending on the severity of the stimulus. The activation of these macroglial cells leads to hypertrophy, which in turn induces the overexpression of vimentin (an intermediate filament) and glial fibrillary acidic protein (GFAP), which is considered a hallmark of retinal stress [96]. As an immediate response to non-permanent acute stimuli, the RMG promotes the secretion of trophic and antioxidant factors, but as it becomes chronic, their secretory role can be clearly deleterious to neuronal cells [96].

Therefore, the abovementioned state of hyperoxia and the ensuing ROS formation are fundamental underlying causes of accelerated rod loss and cone injury in the retina affected by RP.

## 3. Mesenchymal Cells: Therapeutic Strategies in Retinitis Pigmentosa

Over the past few years, different therapeutic approaches aiming to delay the rod death and to prevent the cone injury in RP have been explored. In particular, much emphasis has been placed on cell therapy and gene therapy. The latter one, however, has achieved limited results in vivo and it may not modify the retinal damage once it has occurred. Consequently, scientific interest is particularly focused on cell therapy, a promising tool of regenerative medicine [97].

Some researchers have used embryonic stem cells [98] or induced pluripotent stem cells [99] to generate neurons that could replace lost cells. Although these cells effectively express neuronal markers, most of them show a poor retinal integration, remaining close to the injection site.

Other researchers have used mesenchymal stem cells (MSC) by exploiting their primary ability to paracrinally modulate the neuronal microenvironment by secreting growth factors (GF) in different retinal degeneration models [100,101,102,103,104,105].

Cell therapy can contribute to maintain both the neuronal density and the function of the retina by improving and preserving intra- and extra-cellular conditions [106].

Compared to ESCs and iPSCs, MSCs have a lower differentiation potential, but numerous advantages: they do not induce risks of uncontrolled growth and rejection reactions, not requiring immunosuppressant use; they do not have ethical problems; they are relatively inexpensive and easy to collect (especially those derived from adipose tissue); finally, they have a higher immunomodulatory capacity, meeting the prerequisites of regenerative medicine [107,108,109].

MSCs are characterized by the group of cell surface markers, both positive and negative, proposed by the International Society for Cellular Therapy in 2006 [110]. The MSC population is defined as >95% positive for CD105, CD73, CD34, and CD90, and >95%, negative for CD45, CD14 or CD11, CD79, CD19, and HLA-DR. MSCs also express other surface markers, such as CD44, CD166, Stro-1, CD106, and CD146 [111].

MSCs, spread ubiquitously throughout the body, play a key role in organogenesis, tissue remodeling, and repair [112].

They can migrate to injury sites, following the intravascular administration. This process is due to the distinctive molecules present on the surface of MSCs and endothelial cells, such as P-selectin and integrins [113]. For this reason, these cells have the ability to adhere to the endothelium and cross it by metalloprotease [114].

Among the different sources, the most interesting MSCs exploited for clinical therapeutic purposes in retinal diseases include:

- Adipose-derived stem cells (ADSCs)
- Adult adipocytes
- Platelets

Adipose tissue is one of the most interesting collection sites of MSC. Like bone marrow, adipose tissue contains a large population of stem cells, called ADSCs, within its stromal compartment. They can be obtained using simple procedures such as lipoaspiration performed under local anesthesia. ADSCs are more numerous, have a faster expansion, and a greater secretory and immunomodulatory capacity [109].

ADSCs produce basic fibroblast GF (bFGF) also known as FGF2, vascular endothelial GF (VEGF), macrophage colony-stimulating factor (M-CSF), granulocyte-macrophage colony-stimulating factor (GM-CSF), placental GF (PlGF), transforming GF beta (TGF-β), hepatocyte GF (HGF), insulin-like GF-1 (IGF-1), interleukin (IL), angiogenin, ciliary neurotrophic factor (CNTF), brain-derived neurotrophic factor (BDNF) [108,115], and glial cell-derived neurotrophic factor (GDNF) [116].

Adult adipocytes are another type of mesenchymal cell that can be used for regenerative purposes. These can secrete specific hormones, called adipokines, which play a role in energy homeostasis. Adipose cells produce epidermal GF (EGF), bFGF, IGF-1, IL, TGFβ, pigment epithelium-derived factor (PEDF), and adiponectin [117,118,119,120].

Finally, also the platelets, originating from the subdivision of megakaryocytes, originate from mesenchymal tissue.

They are well known for their hemostatic action, but they can also release substances that promote tissue repair and angiogenesis, and modulate inflammation [121]. In addition, they induce cell migration and adhesion at angiogenesis sites, as well as differentiation of endothelial progenitors into mature endothelial cells [122].

Platelets produce platelet-derived GF (PDGF), IGF-1, TGFβ, VEGF, bFGF, EGF, platelet-derived angiogenesis factor (PDAF), and thrombospondin (TSP), and several authors have used them in eye diseases such as glaucoma, age-related macular degeneration (AMD), and RP [123,124,125,126].

They are used in regenerative therapy in the state of platelet rich plasma (PRP), obtained from plasma centrifugation, because it allows to achieve a greater production of cytokines, even 4–5 times greater than the initial conditions.

Several cell grafting methods have been developed: intravitreal [104,127], subretinal [128], epiretinal, subtenon [126], and suprachoroidal [129,130,131,132] (Table 1). Each has its advantages and disadvantages.

In particular, the suprachoroidal implantation of MSCs according to Limoli Retinal Restoration Technique uses three types of autologous mesenchymal cells: ADSCs, adipocytes, and platelets concentrated in PRP. With this method, improvements have been observed in electroretinographic parameters and visual performance in AMD, opticopathies, and RP. Furthermore, it seems to be devoid of the potential complications reported for the intravitreal and subretinal methods [128,131,132,133,134].

The ocular administration of MSC promotes a significant restoration of the visual system in a variety of eye diseases, including RP [100,135,136,137,138], through several mechanisms, as follows (Table 2):

1. Cell differentiation and trans-differentiation for lost/damaged cell replacement
2. Paracrine action for cell repair and functional stimulation
3. Exosomes and microvesicle secretion
4. Modulation of host immune responses in inflammation site

### 3.1. Transdifferentation

Experimental studies have described the ability of MSCs to differentiate mainly into adipocytes, chondrocytes, osteoblasts, vascular endothelial cells, cardiomyocytes, pancreatic beta cells, and hepatocytes, as well as into retinal progenitor cells, photoreceptors, and retinal neuron-like cells [101,102].

In fact, it has been shown that, in presence of retinal cells, supernatant from retinal cell cultures, or retinal cell extracts, MSCs differentiate into those cells by expressing genes and markers typical of retinal cells [139].

### 3.2. Paracrine Effect

**Figure 1:**
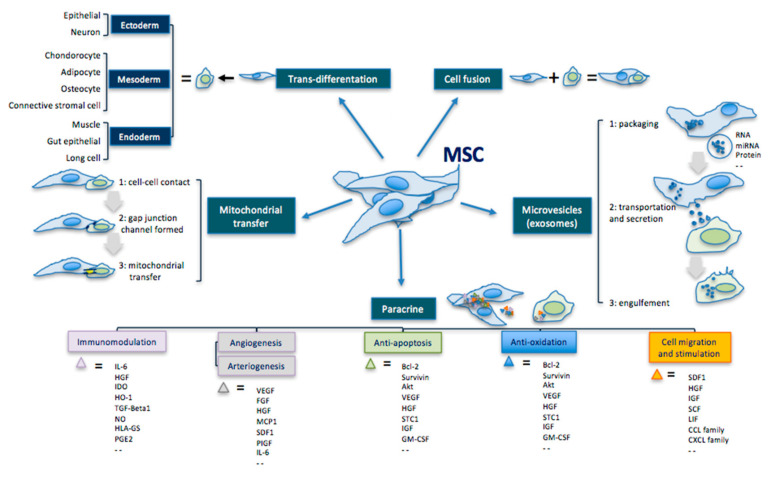
Principal therapeutic mechanisms of MSCs, modified by Liang [156].

In addition to their multipotent differentiation capacity, MSCs can also secrete active ingredients—such as cytokines, chemokines, and GFs—that act paracrinally (Figure 1) [66,140,141,142,143,144,145,146,147,148].

The cytokines produced by mesenchymal cells, after binding with the specific receptor on the target cell, activate specific signaling pathways by phosphorylation processes. Consequently, the transcription factors enter the nucleus and, by interacting with nuclear DNA, regulate the cellular transition from G0 to G1, necessary to activate gene expression and protein synthesis. The end products play a key role in cell survival, including mitosis, migration, and cell differentiation [129,130,131,132,133,149].

The mesenchymal cell graft is the most effective method for therapeutic purposes because it produces a variety of bioactive molecules on an ongoing basis. In fact, although the secreted factors have a short half-life, the cell graft ensures their production continuously, maintaining a long-term therapeutic effect [150,151].

### 3.3. Extracellular Vesicles

Furthermore, growing evidence has been reported on the therapeutic potential of extracellular vesicles and exosomes, that MSCs can release in the extracellular environment [152,153].

Exosomes and microvesicles are very different from each other but have common characteristics in terms of size and content: both carry RNA, proteins, enzymes, and lipids, as well as mitochondria and ribosomes. Through these factors, they can regulate various biological functions, including the repair of damaged tissues [152,154].

Thus, these particles could be directly use for therapeutic purposes, without the need to graft MSCs [152].

Intravitreal injection of MSC-derived exosomes has been shown to exert a repair and protective action in murine models of laser-induced retinal damage [155]. The author has noted that transplanted exosomes inhibit infiltration of inflammation-mediated cytokines, including stromal cell-derived factor 1 (SDF1), monocyte chemotactic protein-1 (MCP-1), tumor necrosis factor (TNF-α), and intercellular adhesion molecule-1 (ICAM-1) and, generally, T-cell-mediated immune responses [155,156].

**Table 2:** Biological actions of MSC-based therapeutic applications [156].

MSC Effects	Mechanisms	Comments
Transdifferentiation	Ability to differentiate into the three germ leyers cells.	Ectoderm: epithelial cell, neuron Mesoderm: condrocyte, adipocyte, osteocyte, connective stromal cell Endoderm: muscle cell, gut epithelial cell, lung cell
Cell fusion	Ability to fuse with another cell forming a heterokaryon (i.e. multinuclear cell).	
Mitochondrial transfer	Ability to transfer mitochondria in damaged cells to increase activity of the respiratory chain complex and ATP levels.	MSC makes contact with the targeted cell and builds a gap junctional channel to transfer mitochondria.
Extracellular vesicles	Ability to release microvesicles and/or exososomes containing bioactive molecules, RNA, microRNA, lipids and proteins for intercellular communication.	The interaction of extracellular vesicles with the targeted cell leads to fusion, release and transfer of the vesicles’ components.
Paracrine effect	Ability to secrete bioactive cytokines and chemokines that act on immunomodulation, angiogenesis/arteriogenesis, antiapoptosis, antioxidation and cell migration/stimulation.	Examples: IL-6; HGF; IDO; HO-1; TGF; NO; HLA-G5; PGE2; VEGF; FGF; IGF; MCP1; SDF1; PIGF; IL-6; Bcl-2; Akt; STC1; GM-CSF; TNF; GDNF; SCF; LIF; CCL; CXCL.

MSC, mesenchymal stem cells; IL-6, interleukin-6; HGF, hepatocyte growth factor; IDO, indoleamine 2,3-dioxygenase; HO-1, heme oxygenase 1; TGF, transforming growth factor; NO, nitric oxide; HLA-G5, human leukocyte antigen class I molecule G5; PGE2, prostaglandin E2; VEGF, vascular endothelial growth factor; FGF, fibroblast growth factor; IGF, insulin-like growth factor; MCP1, monocyte chemotactic protein 1; SDF1, stromal cell-derived factor 1; PIGF, placental growth factor; IL-6, interleukin 6; Bcl-2, B-cell lymphoma 2; Akt, v-akt murine thymoma viral oncogene homolog 1; STC1, stanniocalcin 1; GM-CSF, granulocyte-macrophage colony-stimulating factor; TNF, tumor necrosis factor; GDNF, glial-derived neurotrophic factor; SCF, stem cell factor; LIF, leukemia inhibitory factor; CCL, chemokine C-C motif ligand; CXCL, chemokine C-X-C motif ligand.

## 4. Cell-Mediated Biomolecular and Antioxidative Mechanisms in RP

The therapeutic effect of MSCs is mainly based on the paracrine secretion of cytokines, GFs, extracellular vesicles and exosomes. In recent years, the scientific literature has highlighted the several mechanisms through which the cell therapy can slow down the RP progression. The therapeutic mechanisms are summarized below:

1. Hemorheological activity
2. Antioxidant activity
3. Anti-inflammatory activity
4. Anti-apoptotic activity
5. Cytoprotective activity

It is important to note that the boundaries between these mechanisms cannot be clearly distinguished.

### 4.1. Hemorheological Activity

MSCs can help to regulate retinal microhemodynamics through their paracrine secretome.

In a study conducted on a murine model of diabetic retinopathy, the administration of bone marrow-derived MSCs has been followed by their integration into the retinal structure and their subsequent differentiation into RMG, likely by contact mechanism. Consequently, they may exert selective protection against retinal gliosis and restore vascular integrity and function [85].

In another study conducted on a murine model of diabetes, intravitreal administration of adipose tissue-derived MSCs was not followed by any signs of diabetic angiopathy, such as neovascularization, ischemia, loss of RGC, or increased pro-angiogenic factors, compared to untreated cases [157].

The implanted MSCs secrete a wide range of GFs and cytokines, as well as other proteolytic and angiogenic proteins, including VEGF, bFGF, angiogenin, PDAF, PlGF, PDGF, EGF, TGF-β1, SDF-1, cathepsins, MMP (or matrix metalloproteinases), and PAI-1 (plasminogen activator inhibitor 1) in response to tissue repair [137,158].

In this way, they can promote endothelial regeneration and can thus contribute to boosting microhemodynamics. Through the release of anti-angiogenic factors, such as TSP-1 and PEDF, they can exert an inhibitory action on pathological neovascularization [157,159,160,161].

In 2013, Chu et al. also observed similar suppression in VEGF activity, which was attributed to indirect inhibition of TSP-1 on VEGF receptor through the binding to CD36 and subsequent recruitment of SHP-1 (Src Homology 2 domain-containing Protein tyrosine phosphatase 1), a negative regulator of cell activation and proliferation [162].

Platelets can also release factors—such as PDGF, bFGF, EGF, VEGF, IGF-1, TGF-β, PDAF, and TSP—that promote tissue repair and regeneration and angiogenesis. In addition, they can modulate inflammation and apoptosis, stimulate cell migration and adhesion at angiogenesis sites, and improve the differentiation process of endothelial progenitor cells into mature cells [121].

The platelets can be administered as PRP, promoting new capillary plexus development and facilitating nutrient supply to the grafted cells [163].

Subretinal injection of PRP in a neonatal mouse model has been shown to promote the formation of denser vascular networks [164].

Even ADSCs grafted at an early stage in the subretinal space can prevent the progression of diabetic retinopathy [165].

### 4.2. Antioxidant Activity

As seen above, the oxidative stress has been shown to play a significant role in the pathogenesis of PR and disease progression. Based on the causative gene of RP, antioxidant treatments could preserve the cone function and prolong the rod survival [8,79].

**Figure 2:**
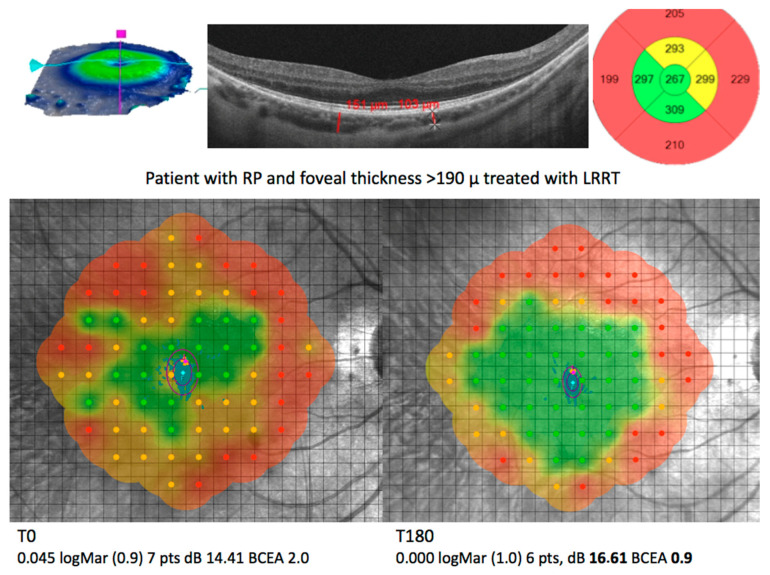
Image showing the effect of the suprachoroidal implantation of autologous mesenchymal cells in a patient with retinitis pigmentosa. The OCT shows the foveal area thickness; the thicker it is, the greater the interactions between growth factors and residual cells. It can explain the increase in visual performance (BCVA, dB, pts) at T180. Image courtesy of P. Limoli—Low Vision research Centre of Milan.

Among the various treatments that can limit oxidative damage, MSCs seem to play an interesting therapeutic role [94,100,159,160,166,167]. The MSC secretome is very wide and different according to the specific experimental context, probably due to the varying ROS inductors. There is evidence that MSC therapy can have a broad influence on the redox context due to these antioxidant factors [168] and it can positively influence the evolution of RP (Figure 2) [131,132].

Indeed, the concentration of bFGF, one of the most effective molecules in promoting photoreceptor survival in a dose-dependent manner, increases within the external retina in response to oxidative stress [169].

The bFGF is physiologically produced by the RMG stimulated by the glial cell-derived neurotrophic factor (GDNF), which is responsible, inter alia, for the nuclear transcription of bFGF.

MSCs can secrete GDNF. The bFGF, together with other factors such as VEGF act by promoting ischemic containment, metabolic recovery, and neuroprotection [170].

MSCs can directly produce bFGF and modulate the anti-oxidative activity, bypassing the non-functioning RMG in RP.

ADSCs release other neurotrophic factors, such as NGF, bFGF, and GDNF, in order to preserve retinal cell survival and reduce oxidative stress damage in the retina [157].

Moreover, in a mouse model of diabetic retinopathy, after MSC transplantation, it has been shown that some of these cells have been integrated into the retinal structure by differentiating into retinal astrocytes, RGC, pericytes, and RMG, exerting selective protection against retinal gliosis [85].

It has been reported that the ciliary neurotrophic factor (CNTF) and brain-derived neurotrophic factor (BDNF) secreted by MSCs can have a neurotrophic action in RGC culture, after inducing an oxidative state [100].

The containment of oxidative stress delays the rod death as well as indirectly influences the cone vitality, since the paracrine secretion of rod cone viability factor (RdCVF) by rods is crucial for cone survival [5,171]. It has been found that, through an antioxidant effect, RdCVF prevents cone death in transgenic rat models rd10 and P23H [78,172].

### 4.3. Anti-inflammatory Activity

The RMG and RPE produce a set of anti-inflammatory factors such as IL-10, IL-11, and TGF-β to counter the proinflammatory state in the retina affected by RP induced by oxidative stress.

These factors are essential for homeostasis and retinal function. However, their action becomes highly insufficient as RP progresses.

Several studies have suggested that MSCs can express many factors with anti-inflammatory, immunomodulatory and chemotactic action through the crosstalk between MSCs and the microenvironment of the damaged area [173,174,175,176].

It follows that the extent of inflammation and damage can also be modulated therapeutically by creating a balance between the production of pro- and anti-inflammatory molecules.

The intravitreal administration of MSCs can have a significant impact on the immune response of the host through secretion of CNTF and BDNF, neurotrophic factors that can promote the downregulation of pro-inflammatory cytokines such as TNF-α, interferon-γ, and interleukin-1β (IL-1β) [118,165,177].

In addition, they have been proven to exert a protective action against retinal cells through the paracrine release of anti-inflammatory GFs such as IL-6, PDGF, NGF, interferon beta (IFN-β) [178], and activation of the prostaglandin E2 receptor (PGE2R) [143].

Cytokines—such as bFGF, M-CSF, GM-CSF, and IL—which are normally released by the MSCs, have an anti-inflammatory function, and recruit macrophages by chemotaxis that help to eliminate intraretinal cell debris [179,180,181].

The TGF-β1, released by MSCs, contributes to promote the cone survival by an immunomodulatory strategy focused on microglia attenuation [182,183,184].

A study conducted by Guadagni et al. [24] has assessed that an integrated microenvironment with GFs can slow down the genetically determined death of photoreceptors while reducing retinal inflammation, and thus can create better conditions for the viability of the overall cell population.

### 4.4. Antiapoptotic Activity

The increased production of ROS in RP damages phospholipid membranes and cellular DNA, leading to apoptosis and photoreceptor death [77,185].

It has been observed that the administration of mesenchymal cells can hinder apoptosis involved in retinal degeneration.

Apoptosis, or programmed cell death, is a process activated by stimuli of different nature (toxic substances, drugs, oxidative stress, ionizing radiations that cause DNA damage, severe stress on the endoplasmic reticulum or mitochondria, as in ischemic conditions) that leads to cellular self-digestion. In particular, the apoptotic cell undergoes a progressive reduction in volume, a fragmentation of its nuclear DNA, and loses contact with adjacent cells. Subsequently, the cell disintegrates into cellular fragments, which, through the activation of phagocytosis mechanisms, are self-digested, completing the apoptotic process.

In the developing nervous system, the presence of GFs—such as NGF, BDNF, and NT-3 and NT-4—is necessary for the survival of neurons. Their lack at the level of certain neuronal population induces their apoptosis. It means that these GFs perform an anti-apoptotic action.

It is also noteworthy that the apoptosis allows to eliminate the excess cells that have not established the right connections during embryonic development [184].

The MSCs can secrete neurotrophic factors that act paracrinally and inhibit the apoptotic process in RP [186]. In fact, they produce proteins—such as Bcl-2, surviving, and Akt—which have apoptosis-inhibiting characteristics [187].

B-cell lymphoma 2 (Bcl-2) is a protein encoded in humans by the Bcl-2 gene, progenitor of the family of Bcl-2 regulatory proteins that regulate apoptosis, through the expression of caspases (by cysteine aspartase), a family of essential enzymes that implement the programmed cell death.

Bcl-2 has an anti-apoptotic action. The association with the protein Bax transforms Bcl-2 into proapoptotic Bcl-2 (Bax).

The relationship between Bcl-2 and its Bax form determines the sensitivity of cells to a pathological stimulus [188]. The prevalence of Bcl-2 expression over Bcl-2 (Bax) prevents the release of caspase activators; therefore, cells are less likely to respond to apoptotic signaling and vice versa [189].

Survivin is a member of the inhibitor of apoptosis protein (IAP) family, which groups together apoptosis-inhibiting proteins. It can inhibit caspase activation, thus resulting in a negative regulation of apoptosis.

Akt also called protein-kinase B or Pkb is a cytosolic protein that plays a key role in the PI3K/Akt pathway. Its effective result is the activation of biochemical pathways that lead to cell growth and resistance to apoptosis.

Tang et al. have detected the downregulation of Bax expression in the ischemic myocardium after autologous MSC transplantation [190].

MSCs can release VEGF, that prevents apoptosis by overregulating the expression of Bcl-2 in the vascular endothelial cells [191].

VEGF also exerts an antiapoptotic action by phosphorylation of FAK (focal adhesion kinase), a critical signal for cell survival that suppress p53-mediated apoptosis, a protein that physiologically participates in eliminating cells with DNA damage [192,193,194].

Through the paracrine release of exosomes and microvesicles, MSCs can transfer different molecular types or organelles for antiapoptotic purposes.

These transfers are particularly evident when potential target cells are damaged or under stress. For example, MSCs have recently been shown to prevent apoptosis in endothelial cells by transferring mitochondria during ischemic stress [195].

Furthermore, it is known that RPE cells [196,197,198], and the RMG [40,199,200] release GFs in the retinal cytosol that have an anti-apoptotic action; the progressive loss of this cells in RP hinders this protective mechanism. However, the implanted MSCs can alternatively release these factors and stimulate survival not only of photoreceptors and ganglion cells [100], but also of RMG and RPE cells.

### 4.5. Cytoprotective Activity

GFs produced by MSCs have been shown to contribute to neuroprotection by regulating photoreceptor metabolic activity, which is physiologically intense but largely compromised in RP [145,201].

In rat models with hereditary retinal dystrophy, it has been reported that MCS can improve visual function: the paracrine release of trophic cytokines by MSCs can promote the clearance of dysmetabolic photoreceptor products by RPE phagocytes [202].

In addition, their cytoprotective action is expressed through the release of numerous different neurotrophic factors.

Platelet-derived growth factor (PDGF) is one important factor secreted by mesenchymal cells with neuroprotective action; it is a regulator of cell growth and division. In particular, PDGF plays a significant role in blood vessel formation, mitosis, and chemotaxis, inducing photoreceptor survival.

The neuroprotective effects of PDGF are comparable to those of the brain-derived neurotrophic factor (BDGF) [203,204].

This result is based on the ability of PDGF and other molecules produced by MSC to activate the PI3K/Akt/mTOR pathway, and thus to upregulate mTOR signaling; the latter one appears to be decreased in several eye diseases [205].

It has also been discovered that MSC transplantation can reduce the damage to the outer segment layer of photoreceptors by promoting both cell regeneration through the paracrine release of hypoxia-inducible factor-1 (HIF-1) and axonal regeneration through growth-associated protein-43 (GAP-43) [206].

Data from a similar study has assessed that neurotrophic factors—such as NGF, bFGF, and GDNF, released by ADSCs—are involved both in maintaining retinal ganglion cell survival, and in reducing stress-related oxidative retinal damage [40].

Another factor produced by MSCs is EGF, which exerts a neuroprotective action on RMG, stimulating their intracellular transcription and bFGF expression [170,207].

The IGF factor, released by MSCs, promotes the synthesis of DNA and RNA as well as the increase of both cellular number and size. IGF can also regulate neuronal growth and development through a variety of processes, such as neurogenesis, myelination, synaptogenesis, dendritic branching, and neuroprotection following neuronal damage. IGF not only facilitates neuronal connections but also inhibits neuronal death [208,209].

In a murine model of hypertonic ischemia followed by retinal reperfusion, Li and colleagues (2009) injected BM-MSC into the vitreous body: 4 weeks later, the treated eyes had an increased number of RGC compared to the untreated eyes. The treated retinas also showed increased expression of bFGF, BDNF, and CTNF [210].

It has been observed that if the binding of neurotrophic factors, in particular NGF, BDNF, and neurotropin-3, to the Trk receptor in its three variants—A, B or C—is prevented, the neuroprotective effect exerted by cell therapy is reduced [211]. In fact, the Trk pathway has an important role in neuron survival and cyto-functional regulation [211].

Finally, the release of exosomes and microvesicles by MSCs represent another cytoprotective mechanism. As aforementioned, these vesicles contain proteins, mRNA, microRNA, lipids, and organelles—such as ribosomes and mitochondria—and allow this load’s transport from one cell to another. Specifically, the transfer of mitochondria to host cells promote the increase of intracellular AMPc, and therefore the energy level [175]. Islam et al. have recently demonstrated the in vivo evidence of this transfer by MSCs [212].

Proteins present in the extracellular BM-MSC vesicles also include signaling molecules such as mitogen-activated protein kinase (MAPK1), an enzyme expressed by the MAPK1 gene, cell adhesion mediators such as fibronectin, and surface receptors such as the PDGF receptor. MSC-derived extracellular vesicles also express regulatory molecules such as transforming GF beta (TGF-β), galectin-1, and programmed death-ligand1 (PD-L1), mediators involved in the processes of differentiation, proliferation, and cell apoptosis [213,214].

## 5. Conclusions

In view of the highlighted influence of MSC secretome on oxidative stress, the MSC graft in retina or adjacent tissues may slow down RP progression [100,101,104,126,128,131,132]: the bioactive factors released by MSCs could exert a trophic effect on photoreceptors, RMG, and RPE cells, so that the rod and cone lifespan could be prolonged.

## Figures and Tables

**Table 1:** Main clinical studies exploiting MSC for therapeutic purposes in RP.

Disease	Cell Source	Delivery	WHO Identifier	References
AMD (GA), RP and ischaemic retinopathy	Autologous BMHSC	Intravitreal injection	NCT01560715 NCT01518127 NCT01518842	[103,104]
AMD (GA), RP, RVO and DR	Autologous BMHSC	Intravitreal injection	NCT01736059	[105]
RP	Autologous ADMSC	Subretinal application	Not registered	[128]
AMD (GA), RP, OA	Autologous ADMSC And PRP	Suprachoroidal application	Not registered	[131,132,133,134]
RP	Autologous PRP	Subtenon injection	Not registered	[126]
RP	Eterologous UC-MSCs	Suprachoroidal application	Ministry of Health 56733164/203	[129]

RP: Retinitis Pigmentosa; AMD: Age related Macular Disease, GA: Geographic Atrophy, OA: Optic Atrophy; DR: Diabetic retinopathy; RVO Retinal Venous Occlusion; BMHSC: Bone Marrow Human Stem Cell; ADMSC: Adipose Derived Mesenchymal Stem Cell; PRP: Platelet Rich Plasma; UC-MSC: Umbilical Cord Mesenchymal Stem Cell.

## Abbreviations

ADSCs	Adipose Derived Stem Cells
AMD	Age Macular Disease
ASCs	Adipose Stromal Cells
BCEA	Bivariate Contour Ellipse Area
BCVA	Best Corrected Visual Acuity
BDNF	Brain-Derived Neurotrophic Factor
bFGF	Basic Fibroblast Growth Factor
BM-MSCs	Bone Marrow Mesenchymal Stem Cells
CASPasis	Cysteine Aspartate-Specific Proteinases
cERG	Cone ERG or Photopic ERG
CNS	Central Nervous System
CNTF	Ciliary Neurotrophic Factor
EGF	Epidermal Growth Factor
ER	endoplasmic reticulum
ERG	ElectroretinoGram
ESCs	Embrionic Stem Cells
GAP-43	Growth-Associated Protein-43
GDNF	Glial Derived Neurotrophic Factor
GF	Growth Factor
GM-CSF	Granulocyte-Macrophage Colony-Stimulating Factor
HGF	Hepatocyte Growth Factor
HIF-1alpha	Hypoxia-Inducible Factor-1alpha
IAP	Inhibitor of Apoptosis Protein
IFN-β	Interferon-β
IGF-1	Insulin-like Growth Factor-1
IL-1RA	IL-1 Receptor Antagonist
IL	Interleukin
IRD	Inherited Retinal Disease
M-CSF	Macrophage Colony-Stimulating Factor
MAPK1	Mitogen-Activated Protein Kinase
MCP-1	Monocyte Chemoattractant Protein-1
MSCs	Mesenchymal Stem Cells
PDGF	Platelet-Derived Growth Factor
PDAF	Platelet-Derived Angiogenesis Factor
PEDF	Pigment-Epithelium-Derived Factor
PGE2R	Prostaglandin E2 Receptor
PI3-K	Phosphatidylinositol-3-Kinase
PlGF	Placental Growth Factor
POS	Photoreceptor Segments
PRP	Platelet-Rich Plasma
PRDX2	Peroxiredoxin 2
RdCVF	Rod Cone Viability Factor
RGC	Retinal Ganglion Cell
RMG	Retinal Müller Glia
ROS	Reactive Oxygen Species
RP	Retinitis Pigmentosa
RPE	Retinal Pigment Epithelium
SOD	Superoxide Dismutase
SVF	Stromal Vascular Fraction
TGF-	Transforming Growth Factor-
TNF-alpha	Tumoral Necrosis Factor—alpha
TSP	Thrombospondin
UPR	Unfolded Protein Response
VEGF	Vascular Endothelial Growth Factor
